# Thalamo-frontal functional connectivity patterns in Tourette Syndrome: Insights from combined intracranial DBS and EEG recordings

**DOI:** 10.1038/s41380-025-03220-9

**Published:** 2025-09-13

**Authors:** Laura Wehmeyer, Juan Carlos Baldermann, Alek Pogosyan, Fernando Rodriguez Plazas, Philipp A. Loehrer, Leonardo Bonetti, Sahar Yassine, Katharina zur Mühlen, Thomas Schüller, Jens Kuhn, Veerle Visser-Vandewalle, Huiling Tan, Pablo Andrade

**Affiliations:** 1 MRC Brain Network Dynamics Unit, Nuffield Department of Clinical Neurosciences, University of Oxford, Oxford, UK; 2 Department of Stereotactic and Functional Neurosurgery, Faculty of Medicine and University Hospital Cologne, University of Cologne, Cologne, Germany; 3 Department of Psychiatry and Psychotherapy, Faculty of Medicine and University Hospital Cologne, University of Cologne, Cologne, Germany; 4 Department of Psychiatry and Psychotherapy, Medical Center – University of Freiburg, Faculty of Medicine, University of Freiburg, Freiburg, Germany; 5 Department of Neurology, Philipps-University Marburg, Marburg, Germany; 6 Center for Music in the Brain, Department of Clinical Medicine, Aarhus University & The Royal Academy of Music, Aarhus/Aalborg, Denmark; 7 Centre for Eudaimonia and Human Flourishing, Linacre College, University of Oxford, Oxford, UK; 8 Department of Psychiatry, University of Oxford, Oxford, UK; 9 Department of Neurology, Faculty of Medicine and University Hospital Cologne, University of Cologne, Cologne, Germany; 10 Alexianer Hospital Cologne, Alexianer Köln GmbH, Cologne, Germany

**Keywords:** Biomarkers, Psychiatric disorders

## Abstract

Thalamic deep brain stimulation (DBS) has shown clinical improvement for patients with treatment-refractory Tourette Syndrome (TS). Advancing DBS for TS requires identifying reliable electrophysiological markers. Recognising TS as a network disorder, we investigated thalamo-cortical oscillatory connectivity by combining local field potential (LFP) recordings from the DBS thalamic target region using the Percept^TM^ PC neurostimulator with high-density EEG in eight male TS patients (aged 27–38) while stimulation was off. We identified a spatially and spectrally distinct oscillatory network connecting the medial thalamus and frontal regions in the alpha band (8–12 Hz), with functional connectivity strength negatively correlated with TS symptom severity. Moreover, reduced thalamo-frontal alpha functional connectivity before tic onset, localised in sensorimotor regions and the inferior parietal cortex, suggests its direct role in tic generation. Importantly, associations with symptoms and pre-tic dynamics were specific to functional connectivity patterns and not evident in the pure power spectra. These findings underscore the importance of investigating electrophysiological oscillatory connectivity to characterise pathological network connections in TS, potentially guiding stimulation-based interventions and future research on closed-loop DBS for TS.

## Introduction

Tourette Syndrome (TS) is a neurodevelopmental disorder characterised by motor and vocal tics, often co-occurring with other neuropsychiatric conditions, including attention-deficit hyperactivity disorder (ADHD) and obsessive-compulsive disorder (OCD) [1]. Unlike other hyperkinetic disorders, tics are typically preceded by a premonitory urge (PMU) and can be voluntarily suppressed for a limited period [2, 3]. It is widely accepted that dysregulations within the cortico-basal ganglia-thalamo-cortical (CBGTC) circuits contribute to the pathophysiology of TS [4]. For patients with TS, deep brain stimulation (DBS) has emerged as a promising and safe treatment option. DBS of various targets within CBGTC circuits, particularly the thalamus, has demonstrated effectiveness in alleviating TS symptoms [5–7]. However, the mechanism of DBS is still not fully understood, although there is a growing consensus that DBS in general may exert its therapeutic effects by modulating network activity within CBGTC circuits [8, 9]. To further advance and personalise stimulation-based treatment approaches for individuals with TS, it is crucial to develop a comprehensive understanding of the fundamental pathophysiological network mechanisms that should be the primary target of intervention.

Previous research has leveraged the unique opportunity offered by DBS to record intracranial local field potentials (LFPs) in patients with TS. These investigations have unveiled pathological low-frequency activity in the thalamus (range: 2–15 Hz) associated with tic severity and tic generation, suggesting its potential as a biomarker for TS [10–20]. While offering valuable insights, research on LFPs in TS is hampered by small sample sizes and predominantly conducted within intraoperative settings, introducing the surgery-induced microlesion effect and anaesthetics or analgesics as confounding factors [21]. Furthermore, two studies have indicated that resting low-frequency power may normalise or simply change over time following DBS [12, 15], limiting the long-term applicability of these findings. Thus, there is a critical need for identifying a reliable and consistent biomarker that persists over the long term after surgery.

Moreover, previous LFP research in TS primarily focuses on power characteristics and a notable gap persists in our understanding of the pathological functional connectivity between distant brain regions in patients with TS [10–20]. Recognising TS as a network disorder, addressing this issue is crucial. Indeed, findings from neuroimaging [22, 23], stereotactic lesions [24], transcranial magnetic stimulation (TMS) research [25], and animal studies [26] have highlighted the important role of CBGTC circuit connectivity in the TS pathophysiology. A powerful approach to characterise functional connections within CBGTC circuits is to assess electrophysiological oscillatory synchronisation between cortical and subcortical regions. This could be achieved by combining intracranial LFP with scalp recordings, such as EEG. However, since most prior LFP studies were confined to the intraoperative setting, the integration of scalp recordings was complicated due to open wounds and sterility [27].

Addressing these limitations in prior LFP research in TS, advanced implanted neurostimulators with brain sensing capabilities, like the Percept^TM^ PC by Medtronic, now enable the recording of LFPs anytime beyond the intraoperative phase [28, 29]. This recently established, groundbreaking capability offers several advantages for research. Not only does it mitigate potential confounding effects from microlesions and enable the assessment of more naturalistic neural activity already subjected to DBS for an extended period, but it also greatly simplifies the integration of LFP with scalp recordings.

To date, no studies have been conducted using the Percept^TM^ PC in patients with TS to record LFPs, either exclusively or in combination with high-density EEG. Our study aims to address this gap by investigating thalamo-cortical oscillatory connectivity patterns in TS patients with implanted thalamic DBS systems, while stimulation was turned off. The principal correlate of functional connectivity investigated in this study is phase synchronisation, referring to the synchronisation of oscillatory phases between different brain regions. Our primary objective is to characterise oscillatory connectivity patterns at rest in terms of spatiality and spectrality and their association with TS symptom severity. Additionally, we intend to assess the impact of voluntary tic suppression on these spatially and spectrally segregated oscillatory connectivity patterns, and to investigate their potential dynamic changes in relation to the tic. These new insights may help to increase our understanding of electrophysiological markers related to TS symptoms and inform future research on closed-loop DBS for TS.

## Methods

### Participants

Eight adult patients with TS who underwent bilateral implantation of DBS electrodes in the medial thalamus, either the centromedian nucleus–nucleus ventrooralis internus (CM-Voi) or ventral anterior/ventral lateral nuclei (VA/VL), at the University Hospital Cologne between 2009 and 2022 were included in the present study. A detailed description of the surgical procedure targeting the CM-Voi in our centre can be found in Baldermann et al. [6] and for the VA/VL target in Huys et al. [30]. Patients were implanted with either quadripolar or directional DBS leads from Medtronic, Minneapolis, USA. All patients received the Medtronic Percept^TM^ PC implantable pulse generator (IPG) either after DBS lead implantation or when the IPG had to be replaced due to battery depletion. Patients were clinically assessed at the time of testing using the Yale Global Tic Severity Scale (YGTSS) [31] and Premonitory Urge for Tics Scale (PUTS-R) [32]. Please note that out of the initial eight patients included in the study, two had to be excluded from analysis due to excessive noise caused by frequent and intense tics, or excessive drowsiness and frequent eye closure during the experiment (Patient 4 & 5 - see Table 1 for individual characteristics of the final patient group). Each patient provided oral and written informed consent. The study was approved by the Ethics Committee of the Medical Faculty of the University of Cologne (No. 21–1351), registered in the German Clinical Trials Register (DRKS00029073), and performed in accordance with the Declaration of Helsinki.

**Table 1:** Patient characteristics.

	Age	Sex	Months since OP	DBS Target	IPG Side	Coordinates	YGTSS		PUTS
							Total	Global	
Patient 1	27	M	26	CM/Voi	Right	L: X: −5,00 Y: −4,00 Z: 0,00 R: X: 5,00 Y: −4,00 Z: 0,00	34	54	49
Patient 2	33	M	85	CM/Voi	Right	L: X: −5,00 Y: −4,00 Z: 0,00 R: X: 5,00 Y: −4,00 Z: 0,00	14	14	30
Patient 3	27	M	3	CM/Voi	Left	L: X: −5,01 Y: −4,00 Z: 0,00 R: X: 5,00 Y: −4,00 Z: 0,00	25	45	41
Patient 6	24	M	3	CM/Voi	Left	L: X: −6,00 Y: −4,00 Z: 0,00 R: X: 6,00 Y: −4,00 Z: 0,00	20	40	43
Patient 7	38	M	164	VA/VL	Left	L: X: −8,00 Y: −6,00 Z: −2,00 R: X: 8,00 Y: −6,00 Z: −2,00	21	41	35
Patient 8	35	M	104	CM/Voi	Left	L: X: −5,00 Y: −2,00 Z: 0,00 R: X: 5,00 Y: −2,00 Z: 0,00	11	36	22

Abbreviations: CM/Voi = Centromedian Nucleus–Nucleus Ventrooralis Internus; VA/VL = Ventral Anterior/Ventral Lateral Nuclei; IPG = Implantable Pulse Generator; L = Left; R = Right; YGTSS = Yale Global Tic Severity Scale; PUTS: = Premonitory Urge for Tics Scale.

### Experimental design

Recordings took place 3–164 months after surgery (64.17 ± 64.69 (SD)), with DBS turned off and medication unchanged. During the experiment, patients were seated comfortably in an armchair. Data was recorded at rest and during the Real-Time Urge Monitor task [33]. At rest, patients were instructed to relax and to keep their eyes open or closed in alternating order for 7–9 min. Only data collected during the eyes-open condition were used for analysis. Patients were asked to avoid voluntary control over tics and express their tics freely during the rest recording. The Real-Time Urge Monitor task consisted of six 5-min blocks with two alternating conditions: a ‘tic-freely’ condition, similar to the rest recording, and a ‘tic-suppression’ condition, where patients were instructed to make their best effort to voluntarily suppress their tics. Moreover, patients were asked to indicate their PMU intensity in real time by moving a mouse on a scale from 0–100% displayed on the screen. The PMU ratings are reserved for future analysis as part of a separate investigation, and the present study focuses exclusively on the rest, suppression, and tic-related data in line with our present study objectives. For the tic-related analysis, time periods surrounding tics in both the tic-freely and tic-suppression conditions were analysed. To increase the number of tics available for analysis, we combined tics recorded during both the rest recording and the tic-freely condition of the Real-Time Urge Monitor task into a single ‘tic-freely’ dataset, as some patients exhibited relatively few tics. Similarly, for the rest analysis, tic-free and movement-free time periods from both the rest recording and the tic-freely condition of the Real-Time Urge Monitor task were combined to represent rest data within a single ‘tic-freely’ condition. For the suppression analysis, these rest periods were compared with movement-free and tic-free time periods from the tic-suppression condition.

Of note, in patient 6, no rest recording could be performed, and only two blocks of the tic-freely and no tic-suppression condition, because tics occurred only very infrequently during the recording, making tic suppression superfluous.

### Data recordings

EEG was recorded from 63 Ag/AgCl (EASYCAP GmbH, Herrsching, Germany) electrodes according to the extended 10–20 system using the ActiChamp amplifier and the BrainVision Recorder software (version 1.23.0003; Brain Products GmbH, Gilching, Germany). EEG data were online-referenced to Cz, and a common ground was employed at FPz. Recordings were performed with a sampling rate of 5000 Hz and all impedances were kept below 10 kΩ. Additionally, EOGs (vertical and horizontal) and four accelerometers (1D acceleration sensors, Brain Products GmbH, Gilching, Germany) attached to the body parts of the most frequent tics were recorded using the same amplifier and recording software. A video with accompanying audio was recorded, which was synchronised with the EEG using the Brain Vision Video Recorder software (Brain Products GmbH, Gilching, Germany). Simultaneously, bilateral LFPs were recorded with the Percept^TM^ PC using the programmer tablet connected to the communicator placed near the IPG. Specifically, bipolar recordings for each hemisphere were performed in the BrainSense Streaming mode selecting the two outer contacts (0 and 3 for 1×4 and SenSight leads) as sensing contacts adjacent to the two middle contacts selected as stimulation contacts (1 and 2 for 1×4 leads; 1[a,b,c] and 2[a,b,c] for SenSight leads). LFPs were recorded while stimulation was turned off after a wash-out period of at least 2 min. Raw LFPs sampled at 250 Hz were low-pass filtered at 100 Hz and high-pass filtered at 1 Hz [34]. To avoid long recording sessions that could potentially lead to data loss or export failures, a new streaming was started for the rest recording and each single block [29]. During the streaming, the real time LFP was closely monitored for the presence of excessive artifacts and gaps in recordings. For later offline synchronisation of EEG and LFP signals, DBS artifacts were induced in both LFP and EEG by briefly turning the stimulation on and off at the beginning and end of each streaming. Finally, the Percept’s JSON files containing the raw LFP data were exported from the programmer tablet for offline signal processing.

### Selection and marking of tic events

The video recordings, accompanied by audio, were manually inspected offline for motor and vocal tics by an experienced clinician or psychologist using the VLC media player with millisecond precision (VideoLan, Paris, France). The audio track was used to identify and mark the occurrence of vocal tics throughout the recordings. The start and end of each detected tic were marked in the EEG time series using Spike2 (Cambridge Electronic Design, Cambridge, UK). To ensure temporal precision, the timing of tics was cross verified using data from EOGs, accelerometers, and EEG. Subsequently, only tics preceded by a tic-free interval of at least 2 s were selected for analysis. Tics occurring in rapid succession with less than 2 s between them were considered part of a single tic sequence. In such cases, the start of the first tic in the sequence was marked as the start, and the end of the last tic as the end of the sequence. The mean number of recorded tics per patient in the tic-freely condition was 34.50 ± 32.13 (SD), with a range of 10–94 tics, and in the tic-suppression condition 32.20 ± 31.67 (SD), with a range of 11–87 tics. Table 2 provides the final tic numbers included in the analysis after signal processing, along with specifications of the predominant tic type for each patient.

**Table 2:** Trial numbers and analysis inclusion overview.

	Final Trial numbers						Included in …		
	Tic				Rest	Supp	Rest Analysis	Supp. Analysis	Tic Analysis
	Free	Supp	All	Tic Types					
Patient 1	23	11	34	simple motor (body twitching), simple vocal (“hm”), complex motor (touching objects), complex vocal (coprolalia), combined simple motor & vocal	57	48	Y	Y	Y
Patient 2	41	29	70	simple motor (mouth & head movements, eye rolling), simple vocal (“hm”)	38	53	Y	Y	Y
Patient 3	91	85	176	simple motor (blinking, face muscle tensing), simple vocal (“hm”), combined simple motor & vocal	51	29	Y	Y	Y
Patient 6	8	ND	8	simple vocal (“uhu”,”lala”,“hallo”)	30	ND	Y	N	N
Patient 7	11	16	27	simple motor (upper body movement, body twitching), simple vocal (“he”, noise), combined simple motor & vocal	109	72	Y	Y	Y
Patient 8	15	5	20	simple motor (body movement, arm & head twitch)	89	13	Y	N	Y

The order of Tic Types indicates the frequency of these tics, from most to least frequent. Abbreviations: Free = Tic-Freely Condition; Supp = Tic-Suppression Condition; ND = Not Done; Y = Yes; N = No.

### Signal processing

Data was pre-processed and analysed offline using custom-written Matlab scripts (Matlab 2023b, The Mathworks, Natick, MA, USA), Spike2 (Cambridge Electronic Design, Cambridge, UK), EEGLAB 2023.1 [35], and Fieldtrip (version 20230118; https://www.fieldtriptoolbox.org/). A detailed description of the signal processing steps is available in the Supplementary Material. In brief, after importing, synchronising, and cleaning the data, it was organised into distinct epoch types: ‘tic epochs’ (capturing time periods surrounding tics from both the tic-freely and tic-suppression conditions), movement-free ‘rest epochs’ (extracted from the tic-freely condition), and movement-free ‘suppression epochs’ (derived from the tic-suppression condition). The final trial numbers for each condition, along with the specification of which patient was included in which analysis, are provided in Table 2. The data were then decomposed from 2–40 Hz, with power extracted for analysis. To quantify functional connectivity between LFP and EEG signals, the phase synchronisation index (PSI) was calculated for each channel combination between the left and right thalamus and each EEG channel. Power and PSI were subsequently grouped into theta (3–7 Hz), alpha (8–12 Hz), and beta (13–30 Hz) frequency bands. The same calculations were applied to reconstructed cortical source signals.

### Statistical analysis

Statistical analyses were performed at group-level on trial-averaged data using custom-written Matlab scripts. In our study, each thalamus was treated as one independent sample. Comparative analyses between conditions were performed using paired *t-*tests (Matlab function t-test) or multi-way analyses of variance (ANOVA) when testing fixed effects of multiple factors (Matlab function anovan). Significant ANOVA effects were followed by post-hoc pairwise comparisons (Matlab function multcompare). The normal distribution of the data was assessed using Shapiro-Wilk tests (Matlab function swtest). Since most of the data were not normally distributed, statistical significance for all statistical tests was determined by non-parametric Monte Carlo permutation tests [36]. Specifically, p-values were derived by comparing the observed test statistic (i.e., F- or t-statistics) or difference between estimates in the case of pairwise comparisons with a distribution of statistics generated from shuffled data, created by randomly permuting the condition affiliation in 10000 permutation iterations. The p-value is then calculated as the proportion of shuffled test values that exceed the original test value, with the threshold for significance being established based on the top 5% of the distribution of permuted statistics. We applied correction for multiple comparisons using the false discovery rate (FDR) at α = 0.05 (Matlab function fdr_bh) or, for time series testing, cluster-based Monte Carlo simulations (MCS, α = 0.05, MCS p-value = 0.001) [37]. For the MCS analysis, p-values over time were binarized according to a threshold α = 0.05, and clusters of continuous significant values were identified. Binarized values were then shuffled within 10000 permutation iterations, generating a reference distribution of maximum cluster sizes for each permutation run. Original cluster sizes were finally compared against this reference distribution, with clusters exceeding the 99.9th percentile being considered significant [37]. Beyond that, to examine relationships between variables, correlative analyses (Spearman’s correlations) or linear regression analysis were performed. All data are shown as mean ± SEM, unless otherwise indicated.

## Results

### A TS-protective thalamo-frontal alpha network at rest

**Fig. 1:**
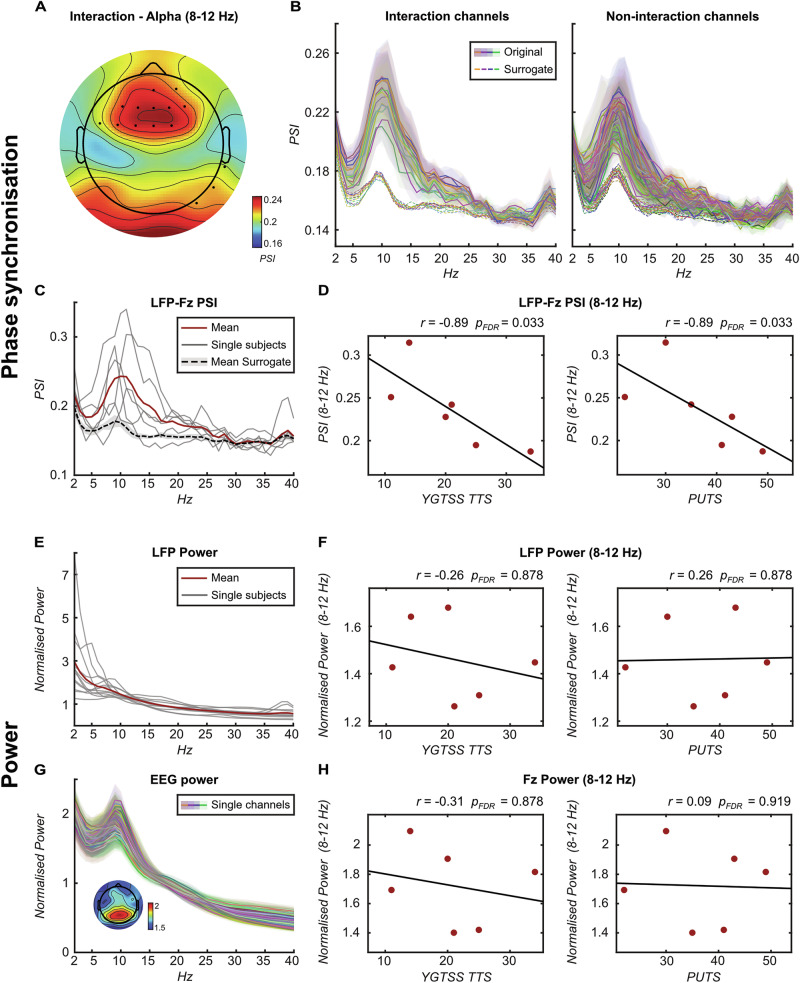
Resting thalamo-cortical connectivity and power patterns. **A** Topographic representation of the PSI calculated over 4-s rest epochs derived from the tic-freely condition between the thalamus and single EEG channels (averaged across subjects) within the alpha frequency range (8–12 Hz). Dots indicate channels with an ANOVA interaction effect, where significant differences between original and surrogate PSI were observed only within the alpha, but not theta or beta frequency range. **B** Frequency plots illustrating original (solid line) and surrogate (dotted line) PSI between the thalamus and single EEG channels (averaged across subjects). Channels with significant interaction effects (corresponding to dots in panel A) are shown on the left, while other channels are displayed on the right. Shading represents standard error. **C** Frequency plot displaying the PSI between the thalamus and Fz, illustrating the original PSI for individual subjects averaged across hemispheres (grey lines), averaged across subjects (red line), and the surrogate PSI averaged across subjects (dotted black line). Shading represents standard error. **D** Scatterplots demonstrating Spearman’s correlations between PSI of thalamus and Fz (averaged across left and right thalamus) within the alpha frequency range and the YGTSS TTS (left) and PUTS (right). **E** Frequency plot showing normalised thalamic LFP power averaged over 4-s rest epochs derived from the tic-freely condition for the individual subjects (grey lines) and the subject’s average (red line). Shading represents standard error. **F** Scatterplots illustrating Spearman’s correlations between normalised thalamic LFP power (averaged across left and right thalamus) within the alpha frequency range and the YGTSS TTS (left) and PUTS (right). **G** Frequency plot presenting normalised EEG power averaged over 4-s rest epochs derived from the tic-freely condition for each channel averaged across subjects with a topographic representation of normalised power for single EEG channels within the alpha frequency range. Shading represents standard error. **H** Scatterplots illustrating Spearman’s correlations between normalised Fz power within the alpha frequency range and the YGTSS TTS (left) and PUTS (right). Abbreviations: YGTSS TTS Yale Global Tic Severity Scale Total Tic Score, PUTS Premonitory Urge for Tics Scale.

To characterise oscillatory connectivity patterns at rest, we conducted a rest analysis involving 6 patients (12 hemispheres), using 4-s rest epochs derived from the tic-freely condition. The normalised power spectrum of the averaged LFP across all hemispheres showed a gradual decrease with increasing frequency, culminating in a visually prominent low-frequency peak (Fig. 1E). In the EEG, dominant alpha oscillations (8–12 Hz) most pronounced over posterior channels were visually observed (Fig. 1G). To determine significant spatially and spectrally distinct thalamo-cortical phase synchronisation patterns, we compared the resting PSI between the LFP and each single EEG channel with surrogate phase-shuffled PSI data across different frequency ranges. For this purpose, we employed a three-way 2 × 3 × 2 ANOVA for each EEG channel, including Condition (original vs surrogate) and Frequency (theta vs alpha vs beta) as within-subject factors, and Hemisphere (left vs right thalamus) as a between-subject factor to account for potential lateralisation effects. We were particularly interested in the interaction effect between Condition and Frequency, as this would indicate that differences in PSI between original and surrogate data vary across frequency bands. Such variation would highlight that certain frequencies show distinct phase synchronisation patterns compared to others, indicating spectral specificity. FDR was applied across all *p*-values obtained for the single EEG channels and pairwise comparisons in case of post-hoc pairwise comparisons. Importantly, an interaction effect between Frequency and Condition was observed for frontal channels. Post-hoc pairwise comparisons revealed a significant difference between original and surrogate PSI for the depicted frontal channels only within the alpha frequency range (8–12 Hz), but not theta or beta (Fig. 1A, B). This finding demonstrates a spatially and spectrally distinct thalamo-cortical phase synchronisation pattern specific to the alpha frequency band in frontal regions. Notably, no significant effects involving Hemisphere were observed, suggesting no lateralisation effects, and justifying the treatment of left and right thalamus as independent samples.

Building upon this, we identified the maximum PSI within the alpha frequency range at Fz (PSI = 0.24 ± 0.02; Fig. 1C), which was also significantly higher compared to the average PSI of all other channels within the same frequency range (*n* = 12, *t*_(11)_ = 3.26, *p* = 0.027, FDR-corrected). Consequently, we designated Fz and alpha as EEG channel and frequency range of interest for subsequent analyses. Next, to explore potential relationships with TS symptoms, we computed Spearman’s correlations between the alpha LFP-Fz PSI averaged across hemispheres and clinical parameters reflecting tic and urge severity (YGTSS total tic score (YGTSS TTS) and PUTS score, respectively), collected at the time of testing. We found a negative correlation between the alpha LFP-Fz PSI and tic/urge severity (*n* = 6; YGTSS TTS: Spearman’s Rho = −0.89, *p* = 0.033; PUTS: Spearman’s Rho = −0.89, *p* = 0.033; FDR-corrected; Fig. 1D), indicating that higher phase synchronisation at rest was associated with less severe symptoms in our patient group. In contrast, no correlations were observed solely for thalamic and frontal alpha power (Fig. 1F, H).

### Absence of a tic suppression effect

To assess the impact of voluntary tic suppression, we performed a suppression analysis involving 4 patients (8 hemispheres), comparing the tic-freely condition with the tic-suppression condition. First, we compared tic frequency between conditions, excluding tics during rest recordings for comparability. No significant difference was found (*n* = 4, *t*_(3)_ = 0.16, *p* = 0.999; Supplementary Fig. 1A). Similarly, when comparing 4-s rest epochs derived from the tic-freely condition with suppression epochs from the tic-suppression condition, we found no effect on either the identified thalamo-frontal alpha phase synchronisation pattern or thalamic/frontal alpha power (LFP-Fz PSI: *n* = 8, *t*_(7)_ = −0.90, *p* = 0.381; Supplementary Fig. 1B; LFP power: *n* = 8, *t*_(7)_ = −0.87, *p* = 0.412; Supplementary Fig. 1C; Fz Power: *n* = 4, *t*_(3)_ = −1.01, *p* = 0.379: Supplementary Fig. 1D). The absence of any tic suppression effect raises doubts about the effectiveness or presence of suppression.

### Tic-related thalamo-frontal alpha connectivity and power dynamics

To gain a comprehensive understanding of general tic-related neural patterns, tic epochs around tic onset from all conditions were pooled for analysis. Because no significant effects of tic suppression were observed and tic occurrence in each condition was limited, no further subgroup analysis was conducted for tics from the tic-freely and tic-suppression conditions.

**Fig. 2:**
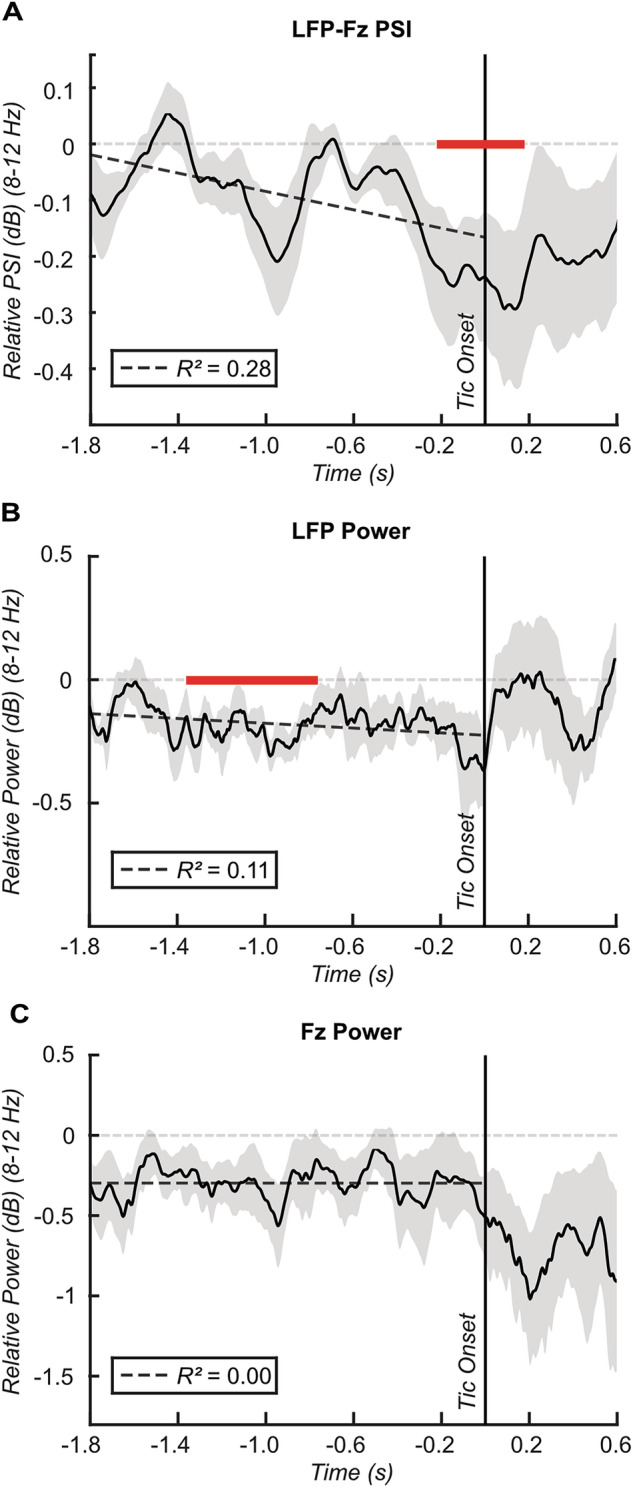
Tic-related thalamo-cortical connectivity and power dynamics at the sensor level. **A** Line plot illustrating the relative change in PSI from rest to tic between the thalamus and Fz within the alpha frequency range (8–12 Hz) averaged across subjects. PSI was calculated within a sliding time window of 0.3 s moving in steps of 0.004 s. Respective PSI values were compared between rest and tic state within a sliding time window of 0.1 s moving in steps of 0.02 s from −1.8–0.6 s relative to tic onset. Red bars indicate time windows of significant difference between tic and rest after cluster-based multiple comparisons correction. A regression line from −1.8 to tic onset depicts the relationship between the relative PSI and time, along with corresponding R^2^ values in the lower left box. Shading represents standard error. **B** The corresponding line plot for relative change in thalamic LFP power. **C** The corresponding line plot for relative change in Fz power.

To capture dynamic changes in thalamo-frontal alpha phase synchronisation around tics, we conducted paired t-tests comparing LFP-Fz PSI values between tic and rest state within a 100-ms sliding time window, moving in steps of 20 ms from −1.8–0.6 s relative to tic onset. Cluster-based multiple comparisons correction revealed a significant PSI reduction from −0.22–0.18 s around tic onset (Fig. 2A). To further explore the observed decreasing trend before tic onset, we performed a linear regression analysis to assess whether temporal changes in the PSI from −1.8 s to tic onset could be explained by time. The results indicated that time leading to the onset of tics accounted for 28% of the variation in PSI (*F*_(1,449)_ = 174.00, *p* < 0.001). Notably, employing the same cluster-based corrected sliding t-test approach to evaluate tic-related dynamics in thalamic/frontal alpha power, we observed a significant reduction in LFP power from −1.36–−0.76 s before tic onset, but no significant changes in LFP or Fz alpha power immediately preceding the tic (Fig. 2B, C). Furthermore, the variance in power before tic onset explained by time was relatively small in case of the LFP (*R²* = 0.11, *F*_(1,449)_ = 58.72, *p* < 0.001) or zero in the case of Fz (*R*^2^ = −0.00, *F*_(1,449)_ = 0.00, *p* = 0.970). While these findings indicate a direct relationship between temporal changes in thalamo-frontal alpha phase synchronisation and tic generation before tic onset, they also suggest that thalamic/frontal alpha power by itself may not play a direct role in tic generation.

**Fig. 3:**
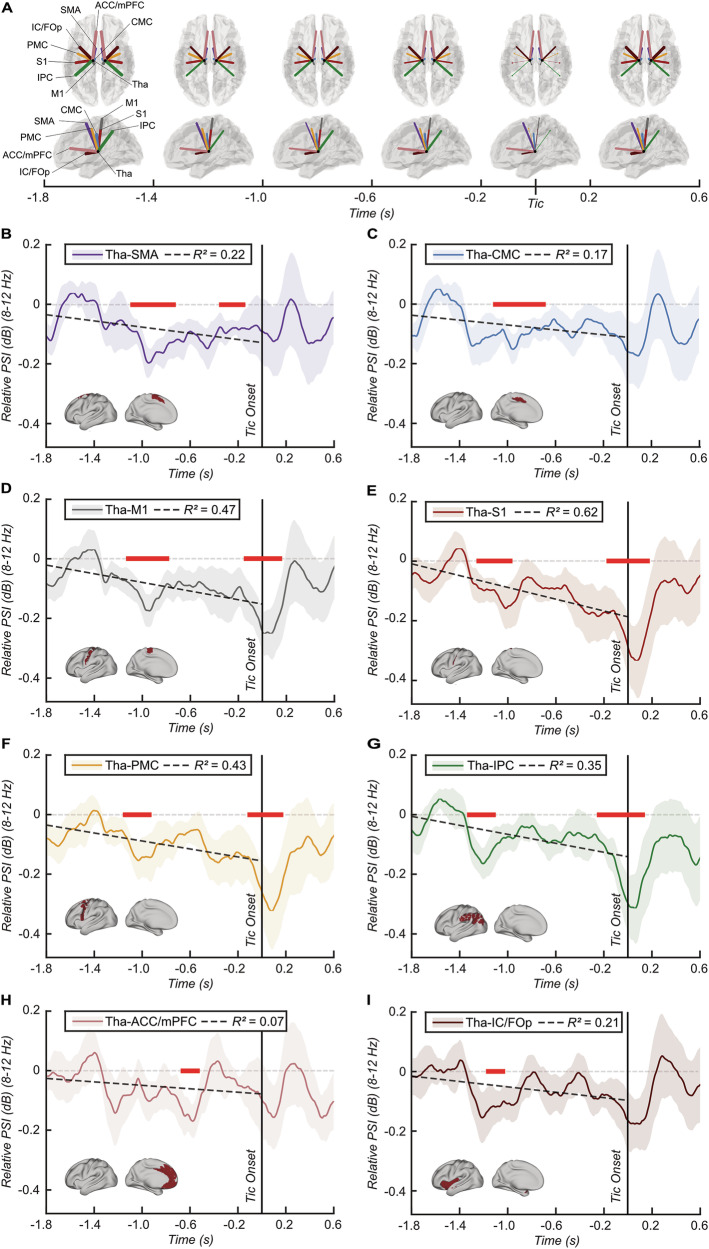
Tic-related thalamo-cortical connectivity dynamics at the source level. **A** Brain templates illustrating the relative change in PSI from rest to tic calculated using the sliding time window approach between the thalamus and eight selected sources of interest within the alpha frequency range (8–12 Hz), averaged across subjects, and aggregated into 400 ms time windows from −1.8–0.6 s around tic onset. Colored lines depict connections between the thalamus and corresponding sources (illustrated for ipsilateral connections in both hemispheres for simplicity), with the thickness of each line representing the strength of the relative PSI in relation to the minimum and maximum PSI values across all sources and time windows. **B**–**I** Line plots showing the time series of the relative PSIs for the respective single sources. PSI values were compared between rest and tic state within a sliding time window of 0.1 s moving in steps of 0.02 s from −1.8–0.6 s relative to tic onset. Red bars indicate time windows of significant difference between tic and rest after cluster-based multiple comparisons correction. A regression line from −1.8 to tic onset depicts the relationship between the relative PSI and time, along with corresponding R^2^ values in the upper box. Shading represents standard error. Brain templates in the lower left corners illustrate the spatial extent of the selected source. Abbreviations: Tha Thalamus, SMA Supplementary motor area, CMC Cingulate motor cortex, M1 Primary motor cortex, S1 Primary somatosensory cortex, PMC Premotor cortex, IPC Inferior parietal cortex, IC/FOp Insular and frontal opercular cortex, ACC/mPFC Anterior cingulate and medial prefrontal cortex.

**Fig. 4:**
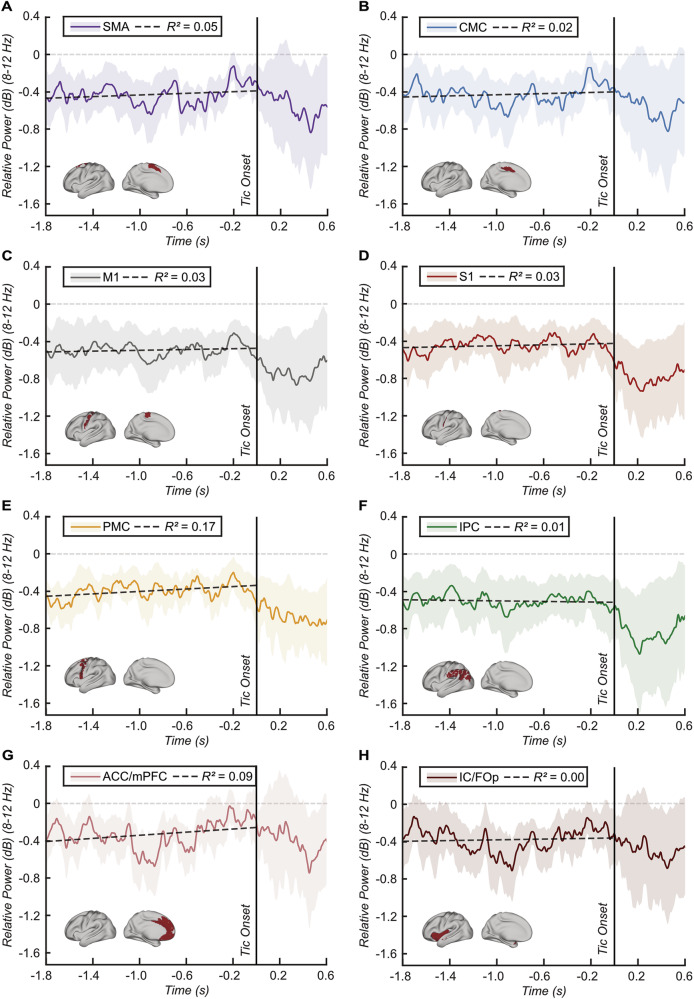
Tic-related thalamo-cortical power dynamics at the source level. **A**–**H** Line plots showing the time series of the relative change in power from rest to tic for each selected source of interest within the alpha frequency range (8–12 Hz) averaged across subjects. Power values were compared between rest and tic state within a sliding time window of 0.1 s moving in steps of 0.02 s from −1.8–0.6 s relative to tic onset. Red bars indicate time windows of significant difference between tic and rest after cluster-based multiple comparisons correction. A regression line from −1.8 to tic onset depicts the relationship between the relative power and time, along with corresponding R^2^ values in the upper box. Shading represents standard error. Brain templates in the lower left corners illustrate the spatial extent of the selected source. Abbreviations: SMA Supplementary motor area, CMC Cingulate motor cortex, M1 Primary motor cortex, S1 Primary somatosensory cortex, PMC Premotor cortex, IPC Inferior parietal cortex, IC/FOp Insular and frontal opercular cortex, ACC/mPFC Anterior cingulate and medial prefrontal cortex.

Expanding our investigation to the source-level, we aimed to better understand the spatial distribution of tic-related temporal changes in thalamo-frontal alpha phase synchronisation. We selected the following eight cortical regions of interest based on observed frontal modulation patterns at the sensor-level and their known relevance to tic generation in TS [2, 23, 24, 38–40]: Primary Motor Cortex (M1), Primary Somatosensory Cortex (S1), Cingulate Motor Cortex (CMC), Supplementary Motor Area (SMA), Premotor Cortex (PMC), Insular and Frontal Opercular Cortex (IC/FOp), Anterior Cingulate and Medial Prefrontal Cortex (ACC/mPFC), and Inferior Parietal Cortex (IPC). Using the same cluster-based corrected sliding t-test approach as described above, we uncovered varied patterns of tic-related temporal dynamics in phase synchronisation between the thalamus and these cortical sources (Fig. 3). Specifically, functional connectivity between thalamus and S1, as well as IPC, showed a brief significant decrease starting around 1.3 s before tic onset (Fig. 3E, G). This was followed by a broader reduction in connectivity involving the SMA, CMC, M1, PMC, IC/FOp, lasting until approximately 700 ms before tic onset (Fig. 3B–D, F, I). Shortly after, a distinct decrease in connectivity to the ACC/mPFC was observed around 600 ms before tic onset (Fig. 3H), followed by another short-lasting connectivity reduction to the SMA around 250 ms before tic onset (Fig. 3B). Finally, connectivity between the thalamus and M1, S1, PMC, and IPC began to decrease again around 200 ms before tic onset, persisting until up to 180 ms after tic onset (Fig. 3D–G). Additionally, regression analyses showed a gradual decrease in connectivity between the thalamus and these regions leading up to tic onset, with up to 62% of the variation in thalamus-S1 PSI changes accounted for by time (*F*_(1,449)_ = 722.16, *p* < 0.001; Fig. 3E). Notably, no significant tic-related source power changes were observed (Fig. 4).

## Discussion

This study represents the first endeavour to combine LFP recordings from the Percept^TM^ PC with high-density EEG, aiming to investigate thalamo-cortical oscillatory connectivity patterns via phase synchronisation in TS patients with an implanted DBS system in the medial thalamus. This innovative approach underscores the practicality of using sensing-enabled neurostimulators, exemplified by the Percept PC from Medtronic, for research purposes, enabling the acquisition of unique data otherwise unattainable, thereby offering invaluable contributions to our understanding of TS. Our findings revealed a spatially and spectrally distinct oscillatory network, connecting medial thalamus and frontal regions in the alpha (8–12 Hz) band, with functional connectivity strength negatively correlated with TS symptom severity. Additionally, we demonstrated a reduction in thalamo-frontal alpha connectivity immediately preceding tic onset, suggesting its involvement in tic generation. Further analysis refining the spatiality of this finding revealed that this modulation extended to sensorimotor regions, including the primary motor cortex, primary somatosensory cortex, premotor cortex, as well as the inferior parietal cortex. Notably, pre-tic-related temporal dynamics are specific to phase synchronisation and not evident in the pure power spectra for both LFP and cortical sources.

Before proceeding to the discussion of our main findings, it is essential to address the absence of a tic suppression effect in the present patient group in this study. While our findings indicate no significant impact on tic frequency from voluntary suppression, we lack additional data related to tic suppression efforts for further insights. Therefore, only speculations can be made about possible explanations. One possibility is that patients did not voluntarily suppress their tics when requested to do so, for various reasons. Some patients may find tic suppression too challenging because they cannot resist the urge [41]. In other patients, symptom severity may have been sufficiently low to render tic suppression superfluous or impractical, given the difficulty in detecting impending tics due to low urge intensity [42]. However, another possibility is that tics were inadvertently suppressed even in the tic-freely condition. Individuals with TS often develop habitual tic control mechanisms, particularly in social settings, implying that tic suppression may occur automatically over time [41, 43, 44]. Consequently, tic control may persist even when patients are not actively attempting to suppress their tics, potentially explaining the lack of differences between the tic-freely and tic-suppression conditions. Therefore, we cannot rule out the potential influence of habitual/automatic tic control in the supposed tic-freely condition. Importantly, even if both habitual/automatic and voluntary tic control resulted in similar tic frequencies, the underlying mechanisms may differ. Our findings show that voluntary tic suppression did not change thalamo-frontal alpha connectivity or thalamic/frontal alpha power, which may indicate that voluntary suppression did not occur, or that both habitual/automatic and voluntary control processes lead to the same effects on these neural patterns. This remains an open question, and we can only conclude that our task manipulation did not have the intended effect. This topic warrants further investigation, as understanding the differences between habitual/automatic and voluntary tic control and their underlying neural mechanisms is crucial for advancing our knowledge of tic control.

Beyond that, it should be noted that the small number of patients (*n* = 4) included in the suppression analysis likely challenged the detection of a tic suppression effect. Furthermore, the heterogeneity of our patient group, which included individuals with very severe symptoms as well as those with minor symptoms due to several months of successful DBS treatment, likely impacts both habitual/automatic and voluntary tic control expression.

Unique to the present study is the comprehensive characterisation of thalamo-cortical functional connectivity patterns across different frequency ranges covering the entire cortex. We discovered a spatially and spectrally distinct thalamo-cortical network in patients with TS at rest, restricted to the alpha frequency band (8–12 Hz) in frontal regions. Notably, simultaneous resting cortical alpha power peaking in posterior, rather than frontal, regions suggests that the observed thalamo-frontal alpha connectivity pattern is independent of overall power activity. Interestingly, we observed a negative correlation between thalamo-frontal alpha connectivity and tic/urge severity. At the same time, no similar correlation pattern could be observed for thalamic and frontal alpha power, emphasising the distinctiveness of the relation between the identified functional connectivity pattern and TS symptomatology. This finding underscores the importance of considering TS as a network disorder characterised by pathophysiological functional connections within CBGTC circuits. It aligns with prior neuroimaging findings of abnormal connections between the thalamus and various frontal regions, encompassing motor and sensory cortices, the cingulate cortex, and the supplementary motor area [22, 45].

The specific mechanism underlying the observed association between increased thalamo-frontal alpha connectivity and reduced symptom severity remains speculative. Building on the earlier notion that habitual/automatic tic control may have been engaged during the tic-freely condition, one plausible hypothesis is that increased thalamo-frontal connectivity could potentially enhance (habitual/automatic) tic control. This is supported by previous research linking fronto-striatal hyperconnectivity as well as general cortical alpha network connectivity to chronic tic control [43, 46]. Also, it has been postulated that tic control involves top-down control mechanisms originating from frontal to subcortical regions, potentially normalising abnormal activity within CBGTC circuits responsible for tics [41, 47, 48]. However, considering that thalamo-frontal alpha connectivity also negatively correlated with urge severity, an alternative or complementary hypothesis could be that increased connectivity may be associated with a reduced PMU. This would also be in line with the observed dynamical decrease of thalamo-frontal alpha connectivity preceding tic execution, as discussed later. Given the thalamus’ role in sensorimotor function as a central mediator of sensory input and perception it is reasonable to posit that thalamo-frontal connections may influence the PMU [49, 50]. Moreover, previous research has highlighted the critical role of frontal regions in the PMU [2, 51, 52].

Although the precise mechanisms are yet to be fully understood, the negative correlation between thalamo-frontal alpha connectivity and symptom severity suggests its potential as a target for stimulation-based treatments in patients with TS. Consistent with this, neuroimaging studies have shown that DBS is most effective when structural or functional connectivity networks linking the thalamus to the frontal cortex, particularly sensorimotor regions such as the (pre-)SMA, cingulate cortex, primary motor cortex, and primary sensory cortex, are stimulated [53–56]. Furthermore, research utilising median nerve stimulation (MNS) highlights the importance of targeting the alpha frequency range, as rhythmic 10-Hz pulse trains have shown significant tic improvement [57, 58]. Rhythmic 10-Hz median nerve stimulation may increase the thalamo-frontal alpha connectivity, which may reduce the occurrence of tics.

Interestingly, we observed dynamic changes in thalamo-frontal alpha connectivity in relation to the tic. These were characterised by distinct functional connectivity decreases between the thalamus and frontal regions, particularly in sensorimotor areas and the inferior parietal cortex, at different timings before the tic. A notable reduction in connectivity around one second before the tic involved various brain regions, including the SMA, cingulate motor cortex, primary motor cortex, primary somatosensory cortex, premotor cortex, inferior parietal cortex as well as the insular and frontal opercular cortex. This indicates that neural processes underlying tic occurrence start well before tic onset, which is in line with the typically observed pre-tic symptomology in TS patients, i.e. the PMU [2]. Furthermore, immediate connectivity decreases around tic onset involved the primary motor cortex, primary somatosensory cortex, premotor cortex, and inferior parietal cortex. This finding is particularly interesting as it implies a direct link to tic generation. Notably, these immediate pre-tic changes were specific to functional connectivity patterns, with no similar direct tic-related dynamic changes detected for mere thalamic or frontal alpha power at either sensor or source level.

Importantly, it should be noted that the tic analysis included tics from both the tic-freely and tic-suppression conditions. Our decision to pool these epochs was driven by the goal of identifying a general tic marker applicable to both conditions. In addition, given the absence of voluntary tic suppression effects, as discussed above, we have no strong reason to believe that the neural dynamics of tics differ between these conditions in the present patient group. However, we must acknowledge that the neural patterns immediately preceding tic onset may differ between the tic-freely condition, which represents usual tics, and the tic-suppression condition, which reflect tics that failed to be suppressed — or potentially not, as it remains unclear whether the tics were actively suppressed, given the absence of significant voluntary suppression effects. Nevertheless, previous studies suggest the potential of a single tic-generation process that is unaffected by voluntary suppression, indicating that all tics, whether attempted to be suppressed or not, might arise from the same fundamental tic-generating mechanism [46, 59]. For this reason, and due to the limited number of tics available, we did not conduct further subgroup analyses comparing these two conditions, and we preliminarily interpret our tic-related functional connectivity pattern as reflecting a general tic-generation process. However, we cannot rule out potential differences between usual tics and those that failed to be suppressed, and we suggest that future studies with a larger dataset explore whether distinct functional connectivity patterns emerge between these states or whether the observed patterns generalize across different tic control contexts.

Our understanding of the precise mechanisms underlying the observed pre-tic disconnections remains speculative. Building on our earlier hypothesis regarding the nature of the resting thalamo-frontal connectivity pattern, the observed disconnection immediately preceding tics might indicate a transient lapse in (habitual/automatic) tic control, potentially facilitating tic execution. However, the temporal pattern of gradually decreasing connectivity over time leading up to the tic suggests more a progressive development of underlying processes, culminating in the manifestation of the apparent tic. Such a process could be more likely related to the PMU, which typically increases before the tic until reaching its peak just before tic onset [2]. It is also plausible that the decreases observed around one second before the tic and immediately before tic onset represent different underlying processes. In fact, the present functional connectivity patterns may stem from a complex interplay of processes involving both tic control and PMU, engaging different brain regions at different timings.

The observed tic-related dynamic functional connectivity changes, encompassing different sensorimotor, frontal, and parietal brain areas are in line with various observations from imaging studies on tic-preceding neural activity [23, 38, 39]. Previous LFP studies have primarily focused on tic-related thalamic power changes, consistently reporting a distinct unrhythmic low-frequency (2–10 Hz) increase following tic onset [10, 13, 14, 16, 18]. Based on this feature, closed-loop DBS approaches in TS have already demonstrated feasibility, safety, and efficacy comparable to continuous DBS [19, 20]. However, these studies did not identify any pre-tic activity changes. Similarly, a recent EEG study found no pre-tic alterations in alpha or beta power in sensorimotor cortices, contrasting with the well-known movement-related beta suppression observed before voluntary movements [60]. This highlights the absence of a distinct electrophysiological power marker preceding tic onset, suggesting the involvement of a complex neural network in tic generation. Prior electrophysiological research on tic-related thalamo-cortical functional connectivity patterns in TS is very limited [10, 18]. One study combining chronic LFP recordings with surface electrocorticogram (ECoG) recordings over the motor cortex detected no thalamo-motor cortex coherence during rest, movement, or tics, which could be related to the limited coverage provided by subdural strips [18]. In another study, intraoperative combined LFP and EEG recordings in three patients revealed repetitive increases in thalamo-cortical coherence preceding tics across broad frequency ranges, including alpha and beta [10]. Discrepancies between these findings and ours may be attributed to factors such as the timing of the recordings and potential cross-subject variability.

In light of this, our results add valuable insights to the existing literature by demonstrating a consistent pattern of pre-tic-related functional connectivity changes across patients, extending beyond the intraoperative time window. These findings may pave the way for future research aimed at identifying electrophysiological pre-tic markers, particularly for closed-loop DBS in TS.

Various limitations of the present study need to be acknowledged. First, it was limited by a small sample size, which restricts the generalisability of our findings to a broader population. Additionally, while the patients exhibited very heterogeneous symptoms, the sample was homogeneous in terms of gender, with all participants being male. We also acknowledge that the small sample size increases the likelihood of spurious correlations, as even strong-looking associations may not replicate or could show different directions in larger samples. Therefore, future studies with larger samples are essential to confirm the reliability and generalizability of these correlations. In addition, while we applied a data-driven approach to focus on Fz for the correlational analyses, the channel showing the strongest connectivity during the rest analysis, we acknowledge that this selection process could introduce bias and inflate the observed correlations. A broader investigation of multiple channels in future studies with larger samples and increased statistical power would help confirm whether these correlations are specific to Fz or generalize across the frontal region. Next, correlation results may be influenced by DBS effects, as clinical parameters reflect symptom severity over the past week when DBS was active. To accurately assess the relationship between thalamic activity and symptom severity in the DBS-Off state, it would be necessary to collect clinical parameters after turning off DBS for at least a week. However, this is unfeasible due to clinical and ethical constraints. A further limitation may arise from potential synaptic plasticity changes following long-term stimulation, especially given the broad range of DBS durations (3–164 months) across patients. The effects of prolonged stimulation might not fully reverse within a wash-out period of 2 min, potentially influencing our results and increasing the heterogeneity in our small sample. In our tic-related analysis, a major limitation is the lack of a control condition for comparison, such as voluntary movements. Additionally, we cannot rule out the potential influence of other movements during the pre-tic state, as patients performed mouse movement as part of the task. The considerable heterogeneity in the phenomenological appearance of tics introduces another limitation, as we were not able to investigate the distinction between vocal and motor tics, which may exhibit different connectivity patterns. However, the current study could not differentiate between these tic types due to the limited number of tics recorded and the presence of combined motor and vocal tics in three out of five patients, as well as the fact that one patient exhibited only motor tics with no vocal tics (see Table 2). It should be emphasised that our primary aim was to identify a common neural substrate underlying tics, irrespective of their specific characteristics. Future research with larger datasets may explore the differences in connectivity between vocal and motor tics, as well as simple and complex tics, more thoroughly.

In conclusion, the present study, combining LFP recordings using the Percept^TM^ PC with high-density EEG in TS patients with thalamic DBS, extends beyond previous intraoperative LFP studies, providing valuable new insights. Our findings implicate the role of a distinct thalamo-frontal network within the alpha frequency band (8–12 Hz) in the TS pathophysiology. Thereby, they underscore the importance of investigating electrophysiological oscillatory synchronisation between subcortical and cortical regions to characterise pathological functional connections within CBGTC circuits. These identified functional connectivity patterns may serve as targets for stimulation-based interventions in TS, informing future research on closed-loop DBS for TS.

## Supplementary information

Supplementary material

## Data Availability

The raw data are not yet openly available, due to data privacy regulations of patient data. We will consider requests to access the data in a trusted research environment as part of a collaboration. Please contact the corresponding authors for this.
