# Supplementary material for: Thalamo-frontal functional connectivity patterns in Tourette Syndrome: Insights from combined intracranial DBS and EEG recordings
Source: Mol Psychiatry. 2025 Sep 13;31(1):231–42. doi: 10.1038/s41380-025-03220-9 (PMC12700786; doi:10.1038/s41380-025-03220-9)
Supplement: Supplementary file 1 — Supplementary material [file 41380_2025_3220_MOESM1_ESM.pdf]

Supplementary material for

Thalamo-frontal functional connectivity patterns in Tourette Syndrome: Insights from combined intracranial DBS and EEG recordings

Laura Wehmeyer, PhD[1,2*], Juan C. Baldermann, MD[3,4], Alek Pogosyan, PhD[1], Fernando Rodriguez Plazas, MSc[1], Philipp A. Loehrer, MD[1,5], Leonardo Bonetti, PhD[6-8], Sahar Yassine, PhD[1], Katharina zur Mühlen, MSc[9], Thomas Schüller, PhD[3,9], Jens Kuhn, MD[3,10], Veerle Visser-Vandewalle, MD, PhD[2], Huiling Tan, PhD[1†], and Pablo Andrade, MD, PhD[2†]

[†] These authors contributed equally to this work.

* Corresponding author: laura.wehmeyer@ndcn.ox.ac.uk

[1]MRC Brain Network Dynamics Unit, Nuffield Department of Clinical Neurosciences, University of Oxford, Oxford, UK

[2]Department of Stereotactic and Functional Neurosurgery, Faculty of Medicine and University Hospital Cologne, University of Cologne, Cologne, Germany

[3]Department of Psychiatry and Psychotherapy, Faculty of Medicine and University Hospital Cologne, University of Cologne, Cologne, Germany

[4]Department of Psychiatry and Psychotherapy, Medical Center – University of Freiburg, Faculty of Medicine, University of Freiburg, Freiburg, Germany

[5]Department of Neurology, Philipps-University Marburg, Marburg, Germany

[6]Center for Music in the Brain, Department of Clinical Medicine, Aarhus University & The Royal Academy of Music, Aarhus/Aalborg, Denmark

[7]Centre for Eudaimonia and Human Flourishing, Linacre College, University of Oxford, Oxford, UK

[8]Department of Psychiatry, University of Oxford, Oxford, UK

[9]Department of Neurology, Faculty of Medicine and University Hospital Cologne, University of Cologne, Cologne, Germany

[10]Alexianer Hospital Cologne, Alexianer Köln GmbH, Cologne, Germany

Methods

Signal processing

EEG data were bandpass filtered (fourth-order Butterworth IIR filter) from 1 to 50 Hz[1] and resampled to 250 Hz to match the LFP sampling rate. The EEGLAB clean_rawdata function was employed to identify flatline, noisy, or outlier EEG channels, with no channels requiring removal. EEG data were then re-referenced to an average reference, and the Cz reference channel was reconstructed using spherical spline interpolation. [2] Raw LFP data were extracted from JSON files using the Percept Toolbox, provided by Thenaisie *et al.* [3], with automatised correction for potential missing data points (available at https://github.com/YohannThenaisie/PerceptToolbox.git). However, LFP data from one tic-suppression block for patient 8 could not be extracted due to transmission problems. The extracted LFP recordings were checked for electrocardiogram (ECG) contamination and, if necessary (n = 1), cleaned of detected ECG artifacts through QRS interpolation using the Perceive Toolbox[4] (available at https://github.com/neuromodulation/perceive). Subsequently, EEG and LFP signals were synchronised for each block/rest recording by manually aligning them with reference to the induced DBS artifacts or termination of high-frequency DBS, if visible in the signal, at the start of the recording. [3] The synchronisation error range was determined by comparing the time difference between the synchronised EEG and LFP signals at the time of the second DBS artifact at the end of the recording. Due to an error range of 260 ms and 1000 ms, one tic-freely block for patient 6, and another tic-suppression block for patient 8 had to be excluded from the analysis. For the remaining recordings, the mean error range was 7.29 ± 6.19 ms (SD) (range: 0 - 32 ms). Next, 5-second epochs were created around each tic onset, containing a 0.5 second buffer at both ends to avoid edge effects after spectral decomposition. Similarly, arbitrary non-overlapping 5-second non-movement epochs were extracted from all recordings by manually selecting time periods during which no tics or other

movements, including mouse movements, occurred, with a minimum 2-second interval from any tic onset/offset. These non-movement epochs represent two distinct conditions: when derived from the tic-freely condition, they reflect pure rest epochs, referred to as ‘rest epochs’, whereas epochs from the tic-suppression condition represent a state of active tic suppression, referred to as ‘suppression epochs’. For rest and suppression epochs, an automated artifact rejection based on extreme values was applied, considering only the time window of interest from -2 to 2 seconds around the arbitrary event [5]. Epochs were rejected if EEG channel amplitudes reached a threshold of ± 200 µV. For tic epochs, each epoch was carefully manually inspected for artifacts in the time window before tic onset. Due to limited numbers of trials, patient 8 had to be excluded from the suppression analysis (n trials = 13) and patient 6 from the tic-related analysis (n trials = 8). The final trial numbers for each condition, along with the specification of which patient was included in which analysis, are provided in Table 2 of the main manuscript. Moreover, an extended infomax independent component analysis (ICA) was run on the segmented data. Independent components labelled as non-brain activity with at least 50% probability according to the IClabel algorithm[6] were subsequently removed after visual inspection of the topographies of the first five components. This resulted in a total of 28.00 ± 8.49 (SD) retained independent components. Next, for each channel, epoched time series were decomposed from 2 to 40 Hz with a frequency resolution of 1 Hz (linear-spaced) using a second order IIR peaking resonator digital filter with a bandwidth of 2 at a 3 dB level, followed by Hilbert transformation. Subsequently, power and phase information were extracted for each epoch from -2 to 2 seconds to cut off the edge effects. Power was normalised by dividing through the mean power from 3 to 40 Hz and averaged in the theta (3-7 Hz), alpha (8-12 Hz), and beta (13-30 Hz) frequency bands.

Phase synchronisation

To quantify the functional connectivity between LFP and EEG signals across time, we calculated the phase synchronisation index (PSI) for each possible channel combination between the left and right thalamus and each single EEG channel. Specifically, the phase difference between signals was computed at all time points for each trial. The PSI over time was then calculated as the vector length of these phase differences within each trial (from -2 to 2 seconds) and subsequently averaged across trials. This yielded an index ranging from 0 to 1, with 0 indicating no synchronisation and 1 indicating perfect synchronisation. Similar to power, the PSI was then averaged in the theta, alpha, and beta frequency bands. Importantly, for the rest and suppression analysis, the PSI was calculated by computing the vector length of phase differences across the entire duration of rest and suppression epochs. Additionally, to determine the significance of phase synchronisation, the PSI of rest epochs solely derived from the tic-freely condition was compared to surrogate phase-shuffled data. The surrogate data were generated 10 times using the same approach as described above, but with the order of LFP trials randomly shuffled, thereby destroying any phase synchronisation, and subsequently averaged. [7] For the tic-related analysis, in order to investigate how cross region functional connectivity changes over time, the PSI was calculated within a sliding time window of 0.3 seconds moving in steps of 0.004 seconds (equal to one data point) from the start to end within each tic and rest epoch (from -2 to 2 seconds), to preserve the temporal dynamics associated with tics.

Source Reconstruction

After preprocessing the EEG data, cortical source signals were reconstructed by solving the inverse problem, using FreeSurfer (available at http://surfer.nmr.mgh.harvard.edu), Brainstorm (available at http://neuroimage.usc.edu/brainstorm), and custom-written Matlab scripts. First, a template MRI (FSAverage) was used to obtain a 3D cortical mesh harboring 15,000 vertices.

[8] Subsequently, the Human Connectome Project (HCP) atlas was mapped to the cortical mesh, and a downsampled parcellation consisting of 26 regions covering each hemisphere was constructed. [9] This process involved merging the 360 cortical areas from the HCP atlas into broader regions, while maintaining fine resolution within regions of the "somatosensory and motor cortex" and "paracentral lobular and mid cingulate cortex," which are particularly relevant to TS based on existing literature. [10-15] Following this, EEG-channels and the template MRI were co-registered by matching individual digitised electrode positions with the surface data from the template MRI. The 3D positions of each EEG sensor on the scalp surface were digitised using an infrared dot-projection 3D scanner, specifically the Structure sensor Mark II (Occipital Inc., San Francisco, CA) integrated with an Apple iPad (8. Gen.). [16] A realistic head model was then constructed using the OpenMEEG toolbox. [17, 18] Finally, a linearly constrained minimum variance beamformer (LCMV) [19] was employed to compute the regional time series data for the 52 cortical regions of interest from the epoched EEG data. In preparation for later tic-related analysis, power extraction and PSI calculation were conducted for cortical sources using the same methodology as for the EEG channels.

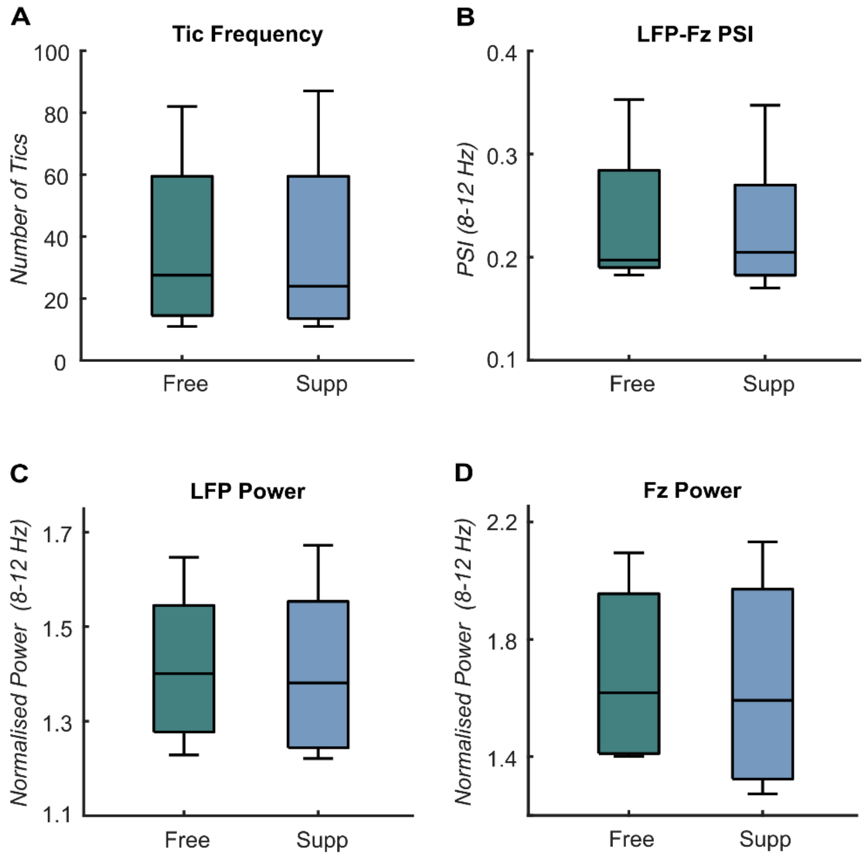

Supplementary Figure 1: Effect of tic suppression. Boxplots showing the group-level difference between tic-freely and tic-suppression conditions for (**A**) The number of tics; (**B**) The PSI calculated over 4-second rest epochs derived from the tic-freely condition and tic-suppression condition between the thalamus and Fz within the alpha frequency range (8-12 Hz); (**C**) Normalised thalamic LFP power averaged over 4-second rest epochs derived from the tic-freely condition and tic-suppression condition within the alpha frequency range; (**D**) Normalised Fz power averaged over 4-second rest epochs derived from the tic-freely condition and tic-suppression condition within the alpha frequency range. Abbreviations: Free = Tic-freely condition; Supp = Tic-suppression condition.

References

1. Oostenveld R, Fries P, Maris E, Schoffelen JM. FieldTrip: Open source software for advanced analysis of MEG, EEG, and invasive electrophysiological data. *Comput Intell Neurosci* 2011; **2011:** 156869.

2. Perrin F, Pernier J, Bertrand O, Echallier JF. Spherical splines for scalp potential and current density mapping. *Electroencephalogr Clin Neurophysiol* 1989; **72**(2)**:** 184-187.

3. Thenaisie Y, Palmisano C, Canessa A, Keulen BJ, Capetian P, Jimenez MC *et al.* Towards adaptive deep brain stimulation: clinical and technical notes on a novel commercial device for chronic brain sensing. *J Neural Eng* 2021; **18**(4).

4. Neumann WJ, Memarian Sorkhabi M, Benjaber M, Feldmann LK, Saryyeva A, Krauss JK *et al.* The sensitivity of ECG contamination to surgical implantation site in brain computer interfaces. *Brain Stimul* 2021; **14**(5)**:** 1301-1306.

5. Delorme A, Sejnowski T, Makeig S. Enhanced detection of artifacts in EEG data using higher-order statistics and independent component analysis. *Neuroimage* 2007; **34**(4)**:** 1443-1449.

6. Pion-Tonachini L, Kreutz-Delgado K, Makeig S. ICLabel: An automated electroencephalographic independent component classifier, dataset, and website. *Neuroimage* 2019; **198:** 181-197.

7. Litvak V, Jha A, Eusebio A, Oostenveld R, Foltynie T, Limousin P *et al.* Resting oscillatory cortico-subthalamic connectivity in patients with Parkinson's disease. *Brain* 2011; **134**(2)**:** 359-374.

8. Desikan RS, Segonne F, Fischl B, Quinn BT, Dickerson BC, Blacker D *et al.* An automated labeling system for subdividing the human cerebral cortex on MRI scans into gyral based regions of interest. *Neuroimage* 2006; **31**(3)**:** 968-980.

9. Glasser MF, Coalson TS, Robinson EC, Hacker CD, Harwell J, Yacoub E *et al.* A multi-modal parcellation of human cerebral cortex. *Nature* 2016; **536**(7615)**:** 171-178.

10. Bohlhalter S, Goldfine A, Matteson S, Garraux G, Hanakawa T, Kansaku K *et al.* Neural correlates of tic generation in Tourette syndrome: an event-related functional MRI study. *Brain* 2006; **129**(8)**:** 2029-2037.

11. Neuner I, Werner CJ, Arrubla J, Stocker T, Ehlen C, Wegener HP *et al.* Imaging the where and when of tic generation and resting state networks in adult Tourette patients. *Front Hum Neurosci* 2014; **8:** 362.

12. Wang Z, Maia TV, Marsh R, Colibazzi T, Gerber A, Peterson BS. The neural circuits that generate tics in Tourette's syndrome. *Am J Psychiatry* 2011; **168**(12)**:** 1326-1337.

13. Ganos C, Al-Fatly B, Fischer JF, Baldermann JC, Hennen C, Visser-Vandewalle V *et al.* A neural network for tics: insights from causal brain lesions and deep brain stimulation. *Brain* 2022; **145**(12)**:** 4385-4397.

14. Rae CL, Critchley HD, Seth AK. A Bayesian Account of the Sensory-Motor Interactions Underlying Symptoms of Tourette Syndrome. *Front Psychiatry* 2019; **10:** 29.

15. Cavanna AE, Black KJ, Hallett M, Voon V. Neurobiology of the Premonitory Urge in Tourette's Syndrome: Pathophysiology and Treatment Implications. *J Neuropsychiatry Clin Neurosci* 2017; **29**(2)**:** 95-104.

16. Shirazi SY, Huang HJ. More Reliable EEG Electrode Digitizing Methods Can Reduce Source Estimation Uncertainty, but Current Methods Already Accurately Identify Brodmann Areas. *Front Neurosci* 2019; **13:** 1159.

17. Tadel F, Baillet S, Mosher JC, Pantazis D, Leahy RM. Brainstorm: a user-friendly application for MEG/EEG analysis. *Comput Intell Neurosci* 2011; **2011:** 879716.

18. Gramfort A, Papadopoulo T, Olivi E, Clerc M. OpenMEEG: opensource software for quasistatic bioelectromagnetics. *Biomed Eng Online* 2010; **9:** 45.

19. Van Veen BD, van Drongelen W, Yuchtman M, Suzuki A. Localization of brain electrical activity via linearly constrained minimum variance spatial filtering. *IEEE Trans Biomed Eng* 1997; **44**(9)**:** 867-880.
